# Monthly Summary

**Published:** 1881-07

**Authors:** 


### MONTHLY SUMMARY.

*The Preservation of the Teeth.*—The following are the directions regarding the care of the teeth issued by the Medical Committee of the National Dental Hospital, London:

1. The teeth should be cleaned at least once a day, the best time being night—last thing. For this purpose use a soft brush, on which take a little soap, and then some prepared chalk, brushing up and down and across. There is rarely any objection to the friction causing the gums to bleed slightly.

2. Avoid all rough usage of the teeth, such as cracking nuts, biting thread, etc., as by so doing even good, sound teeth may be injured.

3. When decay is first observed, advice should at once be sought. It is the stopping in a small hole that is of the greatest service, though not unfrequently a large filling preserves the tooth for years.

4. It is of the greatest importance that children from four years and upwards should have their teeth frequently examined by the dental surgeons, to see that the first set, particularly the back teeth, are not decaying too early; and to have the opportunity of timely treatment for the regulation and preservation of the second set.

5. Children should be taught to *rinse* the mouth night and morning, and to begin the use of the tooth brush early (likewise the tooth-pick.)

6. With regard to the food of children, to those who are old enough, whole meal bread, porridge and milk should be given. This is much more wholesome and substantial food than white bread.

7. If the foregoing instructions were carried out, comparatively few teeth would have to be extracted.

8. Those who do not seek or receive hospital aid are recommended to consult qualified practitioners, and not persons who advertise by show cases, puffing advertisements, etc.

---

*Food for Infants.*—A prize was offered by the Paris Academy of Medicine last year, for an essay on the artificial feeding of infants. The essays show that there is some lack of unanimity in opinion.

The vexed question as to how far the milk should be diluted still remains unsettled, and gastro-intestinal disturbances are variously ascribed to the dilution of the milk or the want of it. In connection with this question, it has been well suggested that milk given fresh from the udder of an animal is to a great extent a living aliment, and much more easy of digestion. Possibly, also, the difference between country and town-fed animals may influence the necessity for the addition of water, and it may be possible to increase the tenuity of the milk by inducing a more frequent drinking on the part of the animals which supply it. No hard and fast rule, however, can be laid down on this subject, but the tolerance of the digestive organs may always be taken as the guide. The addition of a small quantity of sugar is generally recommended, though the possibility of its inducing intestinal derangement is not to be overlooked. A few grains of common salt was proposed by two of the writers. Artificial substitutes for milk were all universally condemned. The importance of most careful supervision of artificial feeding was emphatically insisted upon, and the contrast between its pursuance at home and in public nurseries was shown by the following figures. The mortality of children fed by mothers was 10 to 16 per cent.; by wet nurses, 26 to 30 per cent.; by artificial feeding at home, 15 to 20 per cent.; and away from home, 50 to 60 per cent. The great dangers of the last mentioned system are forcibly dwelt upon.—*Med. and Surg. Reporter.*

---

*Salivary Calculus.*—H. C. Dunavant, M. D., of Osceola, Ark., writes to the *Nashville Journal of Med. and Surg.*, as follows:

The subject of this case, Wm. Payton, (col'd) aged 37 years, now living on my farm in Mississippi County, Ark., hailed me from his cabin door, on the 25tb day of January, last, and requested me to examine his jaw. He said he had been bothered more or less for the last ten years, and during this very severe winter it had given him a great deal of pain.

Upon examination I found the right side of his jaw swollen and hard with a cicatrix on the left side of his jaw, said to have been caused by a gun-shot wound during the late war.

He said there had been some running of pus from under his tongue, and on having him protrude that organ and pull it to the left side, I could see a small opening near the root and right side of his tongue.

Dismounting from my horse and handing his wife my bridle, I proceeded to introduce a small probe and could readily distinguish some solid mass.

Being satisfied that it had no connection with the bone, with a small bistoury I enlarged the opening, and with a pair of common forceps I easily extracted the calculus, which was one inch long by one-half inch wide.

His wife and mother were very incredulous when I told them it was a rock; they said " they had been seten up wid dat boy for de last ten years, makin poltises of hot ashes for his jaw."

Very little hæmorrhage followed its extraction; he was well in a week and says he feels like a new man.

---

*Apparent Insusceptibility to the Influence of Nitrous Oxide.*—Mr. George Myddon read a communication, lately, before the Odontological Society of Great Britain, on the "Behavior of Patients during the Administration of Nitrous Oxide Gas." Of the half dozen cases related the following were the most remarkable: A clergyman, apparently healthy, though he said he suffered severely from dyspepsia, and actively engaged in the duties of his profession, took the gas; he exhibited no signs of nervousness, but inhaled it freely and well. After taking a considerable quantity he declared it was having no effect upon him, and as this was evidently the case, the attempt was abandoned, and the teeth were extracted without the aid of any anæsthetic. Mr. Lyddon estimated that the patient had inhaled about twenty gallons of the gas, but it had no more effect upon him than if it had been common air. The apparatus appeared to be in perfect order; it acted quite satisfactorily with the next patient operated on, and Mr. Lyddon could not give any explanation of the failure. The same thing happened on another occasion, the patient being a lady; in this case, also, anæsthesia could not be induced by nitrous oxide, so chloroform was substituted, with complete success.

---

*Peculiarities of Race.*—The report of Dr. Smith, stationed with the troops at Fort Clark, Texas, shows that there is quite a difference in taste between the white and negro troops at that place. The troops are furnished with certain rations. If they do not care for these they can sell them and purchase other groceries and supplies in their stead. The doctor finds that the negros sold most of their coffee and salt, whence it might be inferred that they did not appreciate these articles. On the other hand, the white troops sold their pork and bacon and pepper. A curious circumstance was the fact that the negro soldier consumes twice as much sugar as a white one; a still stranger fact, the declaration that the darkies use, on the average, three times as much soap as their white brethren. —*Med. and Surg. Reporter.*

---

*Peculiar Obstruction of Wharton's Duct.*—Prof. Richet drew the attention of his class at a recent clinical lecture to a peculiar case of a young man, who, while eating some bread, was suddenly seized with a sharp pain in his tongue, which became so swollen that he could hardly speak. It was supposed that there was a foreign body in Wharton's duct; but it could not be discovered until a few days after, when the patient himself felt a sharp point with his tongue. Nothing, however, could be seen; but soon after a small fragment of straw became dislodged from the duct, and the pain disappeared. He had probably eaten a piece of bread which contained the minute piece of straw.—*Gaz. des Hop. in London Specialist.*

